# New Literature

**DOI:** 10.1155/S146392468000065X

**Published:** 1980


instrumentation. He then introduced Dr D. Deans (ICI Petrochemicals Division) who spoke briefly on the types of visual information provided by graphical displays both for the analyst and the laboratory manager. He particularly emphasised the ability to display analogue information and movement. Dr R. Smith (Loughborough University of Technology) then discussed the uses of graphical display in a wide range of spectroscopic techniques, with particular reference to the display of data, results, operating conditions, statistics and reports. In the ensuing discussion topics considered included interfacing problems, the use of 'touch-screens' for diagnostic purposes, voice inputs, etc.

The main paper on automation was presented by Professor Malmstadt in his plenary lecture on 'Analytical Instrumentation for the 1980's' - he discussed how microcomputers have come to play an increasing role in the laboratory, using as examples some of the work undertaken in his own department.

In conclusion, SAC 80 continued the high standards set by its predecessors both in scientific content and in the range of social activities. It continues to provide an excellent triennial opportunity for analytical chemists to meet their colleagues world-wide. Bearing in mind my earlier comments it will be interesting to see the developments in automation and computerisation over the three years before the next SAC Conference. This reviewer hopes that his remarks on the decline in contributions from the individual laboratory in the field of automation will have proved to be too pessimistic.

*Clive J. Jackson*

### New Literature

### Circulators

In a new bulletin, Fisher Scientific highlights three circulators for circulating temperature-controlled fluids through external apparatus. The model 73 provides ±0.1°C control within its ambient to 150°C temperature range. This immersion circulator can be clamped to the edge of a tank, aquarium or jar-style bath. Designed for close temperature control, the model 80 features an insulated one gallon equilibration bath. Within its ambient to 150°C temperature range, control accuracy is claimed to be ±0.003°C. This circulator can be connected to an auxiliary coolant source for operation at temperatures from below 0°C. Designed for low temperatures model 90 heats or cools small flasks and tubes to temperatures between -15° and 100°C with ±0.02°C accuracy. It is equipped with a built-in refrigeration unit and a one gallon bath. All three units provide thermoregulator control, solid state circuitry, high-capacity pump, stainless steel immersion components and adjustable heater wattage. Details of these circulators are given in Bulletin Number 531.

*Fisher Scientific Co, 711 Forbes Avenue, Pittsburgh, PA 15219, USA.*

### ESR system

The Sterilin Gazette, Number 11, features news on the Accu-Tech ESR (Erythrocyte Sedimentation Rate) system, which is now available in the UK from Sterilin. There is an article on the 'Glassless Revolution' with details on the production of Petri dishes plus information on other products available from this company.

*Sterilin Ltd, 43-45 Broad Street, Teddington, Middlesex TW11 8QZ, UK.*

### Benzene and toluene analysis

A data sheet available from Techmation describes the analysis of benzene and toluene in petrol to ASTM D3606 using a Carle series S analytical gas chromatograph. Application number 159-A uses two columns connected by a backflush valve, and reduces interference from aliphatics. Separation of aromatics is based on their boiling points. The series S uses an inbuilt precision valve controller factory-set for each 159-A instrument. Valve operation is critical, and results in an initial peak representing non-aromatics below C9, followed by benzene, an internal standard, then toluene. Total analysis time is claimed to be six minutes from injection of a 2 μl sample.

*Techmation Ltd, 58 Edgware Way, Edgware, Middlesex, HA8 8JP, UK.*

### Interface system

A new publication from Bio-Rad Laboratories outlines the use of the CIRA 101 GC/IR interface, coupled with its solid sample accessory, and demonstrates the possibilities of the system by detailing the analysis of the solvent system used in a commercial stamp-pad ink. The experimental detail is fully described and specifies all the chromatographic conditions, together with a typical chromatogram and infra red spectra of the five components used in the solvent system. Concentrations of the components range from 1.2% water to 21% triethylene glycol.

*Bio-Rad Laboratories Ltd, Caxton Way, Holywell Industrial Estate, Watford, Herts, WD1 8RP, UK.*

### Equipment up-date

The current issue of Fisher Scientific's Laboratory Reporter (number 18:1) highlights the latest developments in laboratory hardware, software and technique. It features: the Jet-Clean washer —a programmable unit for cleaning most laboratory glassware; the coal analyser — a self-contained, microprocessor-controlled instrument that can analyse a batch of 80 samples for residual moisture, volatile matter, ash and fixed carbon; and a new microcentrifuge that can handle sixteen 250 μl to 1.5 ml tubes at speeds up to 13750 rpm. The issue also gives details on HPLC-grade water and newly formulated reagents for the sulphur analyser plus convenience supplies such as French curves, an adhesive cement and transparent storage boxes.

*Fisher Scientific Co, 711 Forbes Avenue, Pittsburgh, PA 15219, USA.*

### Waveform processing systems

Datalab has produced a leaflet about waveform processing systems. This features a photograph of the system and a definition of the concept of waveform processing. It explains that the essential elements are a transient recorder to capture analogue signals in digital form, and a backing store such as a magnetic tape recorder to store the digital records for subsequent recall and replay in analogue form. The leaflet also shows a 19 channel transient recorder, associated with a magnetic tape recorder and a CBM model 3032 computer. The features of a system without the computer are listed and then the additional facilities made available by the addition of the computer are set out. This is followed by three examples of typical displays by the computer.

*Data Laboratories Ltd, 28 Wates Way, Mitcham, Surrey, CR4 4HR, UK.*

### Clinical diagnostic products

Bio-Rad Laboratories have published a catalogue for their range of clinical diagnostic products. The catalogue details the company's complete range of test systems including radioassay systems for thyroid, anaemia and drug monitoring; chromatography techniques for catecholamines, porphyria, lead poisoning and diabetes management; enzyme systems for lipid chemistry; together with a complete range of human based sera and urine controls.

*Bio-Rad Laboratories Ltd, Caxton Way, Holywell Industrial Estate, Watford, Herts, WD1 8RP, UK.*

